# Health care effects and medical benefits of a smartphone-based diabetes self-management application: study protocol for a randomized controlled trial

**DOI:** 10.1186/s13063-022-06248-2

**Published:** 2022-04-11

**Authors:** D. Ehrmann, V. Eichinger, I. Vesper, J. Kober, M. Kraus, V. Schäfer, N. Hermanns, B. Kulzer, S. Silbermann

**Affiliations:** 1 Research Institute Diabetes-Academy Mergentheim (FIDAM), Johann-Hammer-Str. 24, 97980 Bad Mergentheim, Germany; 2 mySugr GmbH, Trattnerhof 1/5 OG, 1010 Vienna, Austria; 3 Roche Diabetes Care GmbH, Sandhofer Straße 116, 68305 Mannheim, Germany; 4 Roche Diabetes Care Deutschland GmbH, Sandhofer Straße 116, 68305 Mannheim, Germany

**Keywords:** Diabetes, Self-management, Diabetes distress, Digital health application

## Abstract

**Background:**

Diabetes self-management is a mainstay of diabetes care, but the implementation of self-management regimens into daily life is complex and often results in discouragement and distress. Modern approaches such as smartphone-based self-management applications are therefore needed to support people with diabetes. Since reimbursability would increase the availability of such digital applications to people with diabetes, we designed a study that meets all scientific and methodological requirements set by the German Digital Healthcare Act to allow reimbursement for a specific application (mySugr PRO). Here, we report the protocol of this study that aims at evaluating the efficacy of the digital self-management application with regard to patient-reported outcomes and medical benefits.

**Methods/design:**

This multicenter, open-label, randomized, parallel-group, controlled trial will evaluate the health care effects and medical benefits of mySugr PRO. A total of 466 people with diabetes will be randomly allocated (2:1 randomization) to the interventional group (*n* = 311) that will use the digital self-management application during the 12-week study period or the control group (*n* = 155; no usage of the application). Baseline and follow-up examinations will assess diabetes distress as the primary endpoint as well as empowerment, HbA1c, blood glucose data, self-management, general well-being, and treatment satisfaction as secondary endpoints. Statistical analyses will use an intention-to-treat procedure (using multiple imputation for missing values) as well as a per-protocol approach for sensitivity analysis.

**Discussion:**

To the best of our knowledge, this study will be one of the largest diabetes-specific evaluations of a digital health application supporting people with diabetes in their diabetes self-management that follow the requirements of the German Digital Healthcare Act.

**Trial registration:**

German Clinical Trial Register DRKS00022923. Registered on 22 October 2020.

## Background

Diabetes self-management, the active engagement in a healthy lifestyle and preventive behaviors, is a mainstay of diabetes care. In addition to regular glucose monitoring and treatment adherence, self-management includes the careful planning of diet and physical activities as well as coping with low and high glucose levels [1, 2]. An effective diabetes self-management helps to maintain tight glycemic control and, thereby, to reduce the risk for diabetic complications [3–5]. However, the implementation of diabetes self-management regimens into daily life can be complex and demanding, leading to discouragement and distress [6]. Diabetes-related distress has been proven associated with compromised diabetes self-care [7], reduced health‐related quality of life [8], and impaired glycemic control [9, 10]. Approximately one-third of people with diabetes suffer from elevated diabetes distress [11–13]. This underlines the need for strategies and interventions to support the self-management of people with diabetes [2, 14].

Since people with diabetes are commonly facing constantly changing internal and external influences affecting their glucose levels, tools to support diabetes self-management are required to be permanently accessible to patients. Therefore, digital applications are increasingly employed for diabetes care [15], and meta-analyses demonstrated the beneficial effects of digital applications on weight development, treatment adherence [16], diabetes distress [17], and HbA1c levels in people with diabetes [18, 19].

Reimbursability would make digital applications for diabetes self-management more widely available. In Germany, the Digital Healthcare Act (2019; [20]) requires digital applications applying for reimbursement to be listed in an official register maintained by the German Federal Institute for Drugs and Medical Devices (BfArM), as reviewed by Gerke et al. [21]. To be listed, digital applications need to demonstrate one or more positive health care effects. For diabetes care, these effects could comprise direct medical benefits (glucose, HbA1c, quality of life) and other patient-relevant improvements such as reduced diabetes distress and increased treatment adherence. Methodically, the evaluation of positive health care effects requires a retrospective or prospective comparative study demonstrating better outcomes in patients using the relevant digital application than in patients not using it [21].

One digital health application for people with type 1 or type 2 diabetes is the mySugr PRO app. It was developed in accordance with the requirements for quality management systems for medical devices [22] and has been shown to be positively associated with higher self-care behavior [23]. Data assessed by the app include current glucose levels (automatic upload from monitoring devices is possible), estimated HbA1c (eHbA1c) values, data on medication/insulin intake, and entries on diet, weight, blood pressure, and activity. To add individual context to these medical data, additional information on, for example, time, location, and stress level (general stress, disease) can be entered. Users are then presented with analyses on the eHbA1c and the percentages of hypo- and hyperglycemic values. Traffic light colors indicate critical glucose data, and algorithms for pattern detection identify areas where self-management should be improved. Importantly, concepts known from behavioral psychology such as challenges and direct positive feedback are integrated to foster motivation [24, 25].

As required by the BfArM guidance document [26], a retrospective systematic analysis of glucose metrics from German-speaking app users including a comparison of the app users with a historical cohort of non-users was conducted beforehand to justify the assumed clinical benefits on glycemic control and diabetes distress that are to be confirmed by this study [27]. A more sophisticated study is now required for reimbursement purposes by the BfArM.

Here, we report the study protocol of a parallel, randomized, controlled study to assess the efficacy of the digital health application for diabetes self-management. Efficacy will be assessed via patient-reported outcomes and medical benefits. Study endpoints will comprise the reduction of diabetes distress after 12 weeks (primary endpoint) as well as effects on self-management and treatment adherence, empowerment, self-efficacy, treatment satisfaction, and clinical changes of HbA1c, blood glucose, and quality of life. This study was designed to meet all requirements set by the Digital Healthcare Act and is being conducted between October 2020 and August 2022 in approximately 50 diabetology centers in Germany.

## Method/design

### Study design

This study is a multicenter, open-label, randomized, parallel-group, controlled trial to evaluate the health care effects and medical benefits of a smartphone-based self-management app. In the interventional group, people with diabetes will use the mySugr PRO app as a digital health application during the 12-week study period. The app aims at supporting people with diabetes in their day-to-day self-management but makes no treatment recommendations. The functions of the intervention are summarized in Table 1. In the control group, people with diabetes will continue with their usual mode of therapy, data documentation, and glucose monitoring.

**Table 1:** Summary of mySugr PRO functions

Category	mySugr PRO functions
Data entry	Blood glucose (self-monitored blood glucose [upload], HbA1c) Medication (insulin, other medications) Health data (diet, weight, blood pressure, activity) Context data (time, location, well-being, pictures)
Analysis	Time-course of blood glucose levels Estimated HbA1c (eHbA1c) Hypo- and hyperglycemic episodes Critical glucose data (traffic light colors) Areas for self-management improvement
Motivation	Blood glucose monitoring reminder Game-mechanics: challenges Direct positive feedback loops on self-management success
Patient-physician interaction	Statistics and detailed logs in different data formats

Approximately 50 study centers will take part in this study with approximately 9 participants each. The primary endpoint is the reduction of diabetes distress after 12 weeks. Secondary endpoints comprise diabetes self-management, empowerment, general well-being, self-efficacy, treatment satisfaction, and blood glucose and HbA1c levels. As an additional outcome measure, the real-life experience (e.g., daily helpfulness) of the investigational group will be evaluated by using the ecological momentary assessment (EMA, [28]). The study protocol was prepared in accordance with the checklist for protocols of interventional studies [29] and the DIN standard for the clinical investigation of medical devices for human subjects [30]. The SPIRIT reporting guidelines have additionally been applied [31]. The Ethics Committee of the Medical Chamber of Baden Württemberg has approved the Clinical Investigation Plan of the study on 22 October 2020 (FI-A-1–2020). The study is registered at the German Clinical Trial Register (DRKS00022923).

In sum, the study is designed to evaluate three hypotheses: Compared to a treatment-as-usual control group, the smartphone-based diabetes self-management app is effective in the following:

1. Reducing diabetes distress
2. Improving glycemic control
3. Improving other patient-reported outcomes indicating improved diabetes management

### Study population

People diagnosed with type 1 diabetes (ICD-10 code E10), type 2 diabetes (ICD-10 code E11), or gestational diabetes (ICD-10 code O24.4) are eligible for this study. The inclusion criteria further comprise the capability for both daily blood glucose measurements and corresponding adjustment of therapy, age ≥ 16 years, last obtained HbA1c value < 12% (107.6 mmol/mol), possession of a smartphone compatible with the intervention (Android ≥ 5.0; iOS ≥ 11.4), willingness to accept terms of conditions of the app, German language skills, and provision of informed consent. Patients will be excluded if they are currently using continuous glucose monitoring (CGM) systems (real-time and intermittently scanned CGM), if they used digital diabetes diaries or digital applications for therapy documentation in the last 3 months before study entry, if they have any medical conditions contradicting with diabetes therapy and study participation, or if they are simultaneously participating in another clinical trial.

For the primary outcome, the calculation of the intended sample size was based on the assumption of an expected effect size of 0.3 standard deviations. Statistically, this assumption was derived from a previous analysis evaluating the effect of the digital health application on diabetes distress [27]. Clinically, this effect size was chosen to allow interpretation of clinically meaningful effects as effect sizes below 0.3 have limited implications for clinical practice. Employing a 2:1 randomization, power analysis revealed that 396 participants would be required to achieve a power of 1 − *β* = 0.80 with a two-sided *α* error of 0.05. With an assumed drop-out rate of 15%, we intend to recruit 466 participants (311 intervention, 155 control). Drop-out will be defined as participants who completed baseline assessment of the primary endpoint but were lost to follow-up or withdrew informed consent.

### Randomization, sequence generation, and blinding

Randomization will be performed by a computer-generated randomization sequence (Research Randomizer [32]), assigning participants to the interventional or control group in a 2:1 ratio, by block randomization with an initial block size of six and three for subsequent participants. Study centers will receive concealed envelopes with the randomization results for each participant separately. The respective envelope will be opened by the study staff at the first study visit of the participant, and the participant will be allocated accordingly. Due to the nature of the intervention, blinding of participants and study centers providing the intervention will not be possible.

### Procedures

**Fig. 1:**
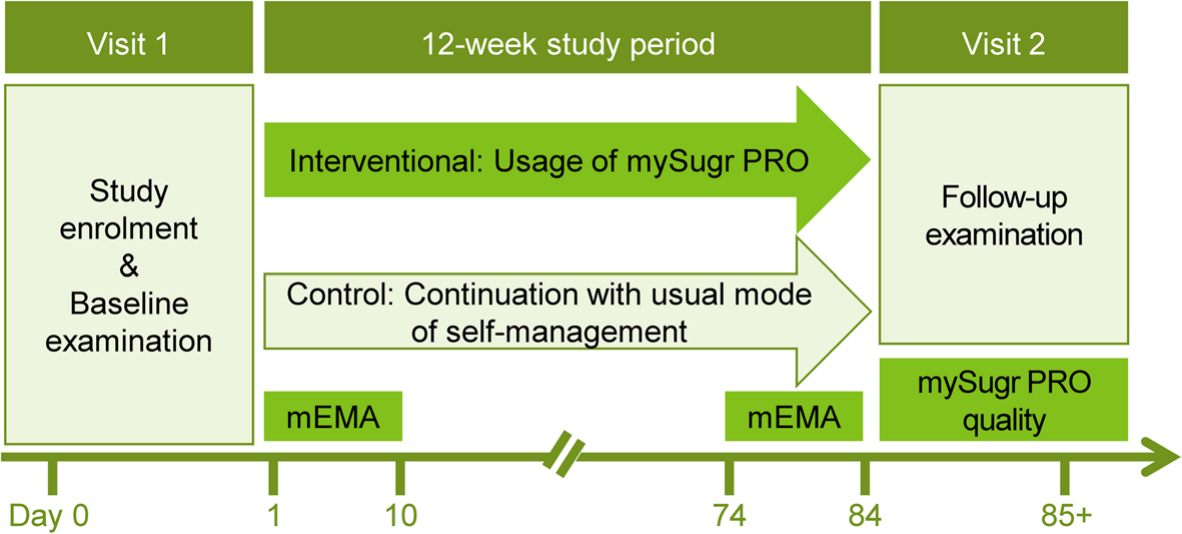
Study visits and procedures. At baseline and follow-up examination, blood glucose data will be collected, and questionnaires on patient-reported outcomes will be completed by all participants. Participants of the interventional group will further report their real-world experience with mySugr PRO by using an application for mobile ecological momentary assessments (mEMA) and by completing an additional questionnaire on the perceived quality of mySugr PRO at visit 2

As depicted in Fig. 1, our study will comprise a 12-week study period that starts and ends with study visits for baseline and follow-up examinations. At study visit 1, participants will be allocated to the study groups according to randomization after informed consent is given. Blood glucose meters from all participants will be read out. Participants of the interventional group will download both the mySugr PRO application (mySugr GmbH, Austria) and the application for mobile EMA capturing time-varying real-life experience (mEMA, ilumivu, USA). Applications will be used via pseudonymized study accounts. Baseline examination at visit 1 will then involve the collection of blood glucose data and the completion of questionnaires on patient-reported outcomes (PRO, see below). Blood glucose data comprise the self-monitored average glucose levels, the corresponding percentages of hypoglycemic (< 70 mg/dl; < 54 mg/dl), euglycemic (70–180 mg/dl), and hyperglycemic (> 180 mg/dl) values, as well as the last documented or routinely assessed HbA1c. During the 12-week study period, participants of the interventional group will use mySugr PRO for diabetes self-management, while control patients will continue with their present mode of self-management. Additionally, participants of the interventional group will daily report their real-world experience via mEMA, both at the beginning and at the end of the interventional phase over a period of 10 days each. Follow-up examination at visit 2 will assess the same data as the baseline evaluation at visit 1, extended by an additional questionnaire on the perceived quality of the intervention and treatment satisfaction (interventional group). Adverse events and serious adverse events will be documented throughout the study. There will be no provisions for ancillary and post-trial care, and there will be no post-trial compensation since we expect no harm due to trial participation.

### Patient-reported outcomes

**Fig. 2:**
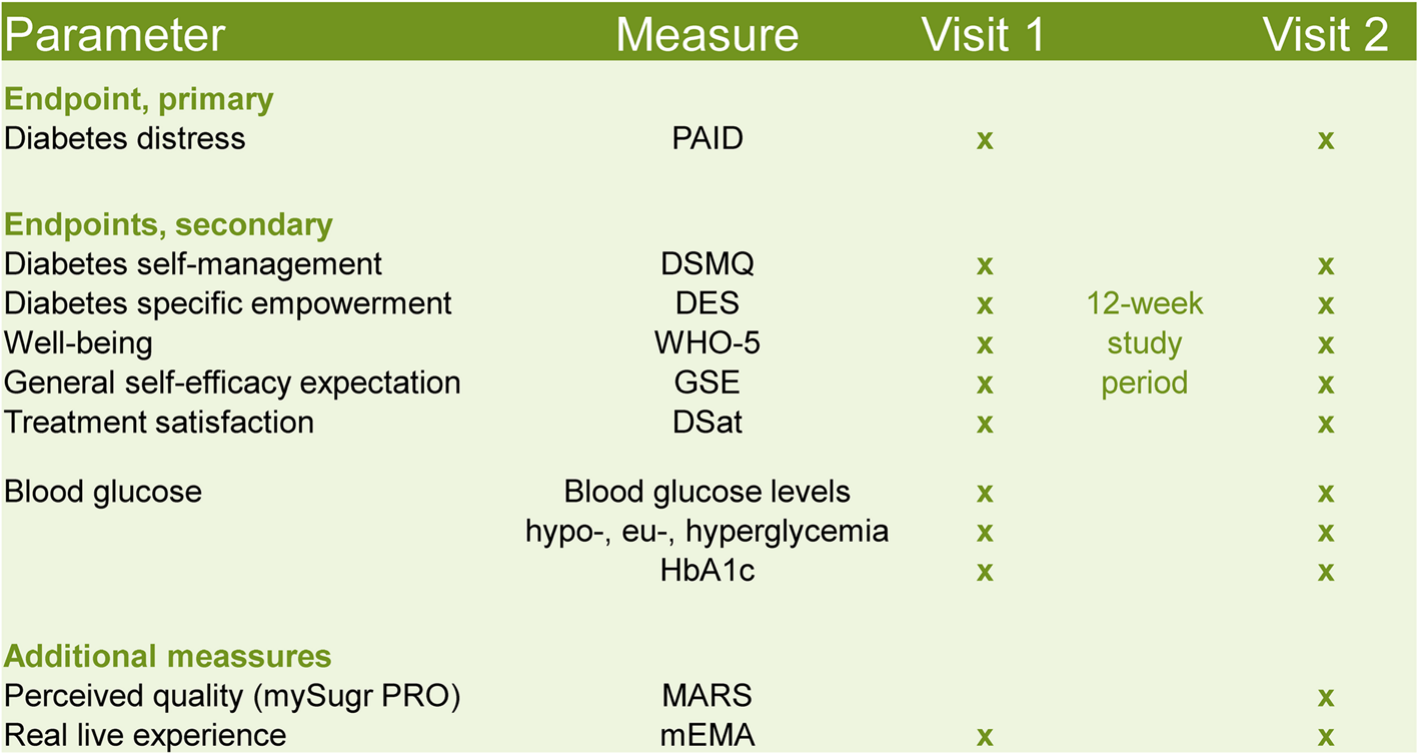
Primary and secondary endpoints and additional measures. PAID, Problem Areas In Diabetes [33]; DSMQ, Diabetes Self-Management Questionnaire [34]; DES, Diabetes Empowerment Scale [35]; WHO-5, well-being scale [36]; GSE, general self-efficacy [37]; DSat, treatment satisfaction [38]; MARS, Mobile Application Rating Scale [39]; mEMA, mobile ecological momentary assessments

At visits 1 and 2, all participants will complete the following PRO questionnaires. As the primary endpoint, the Problem Areas In Diabetes (PAID) questionnaire contains 20 items on areas of diabetes-related psychosocial distress to be rated on a 5-point Likert scale [33]. As secondary endpoints, questionnaires on diabetes self-management, diabetes-specific empowerment, general well-being, self-efficacy expectation, and treatment satisfaction will be completed at visits 1 and 2. In detail, the Diabetes Self-Management Questionnaire (DSMQ) presents a 16-item self-report scale on glucose management, dietary control, physical activity, and health care use, with each item to be rated on a Likert scale ranging from 0 (does not apply) to 3 (very much applies) [34]. The questionnaire on diabetes-specific empowerment bases on the German version of the diabetes empowerment scale (DES; 11 items to be rated on a 4-point scale; [35]). General well-being will be assessed by the WHO-5 scale, in which patients will be required to rate how well each of five statements applies when considering the last 14 days [36]. The questionnaire on general self-efficacy (GSE) contains 10 items to be rated on a 4-point Likert scale [37]. For the evaluation of diabetes treatment satisfaction, we used the questionnaire for the assessment of diabetes-related problems and satisfaction with insulin treatment (DSat; [38]). The DSat questionnaire has 10 questions, each of which is scored on a scale ranging from 1 (very satisfied) to 6 (completely dissatisfied). The topics of this questionnaire include satisfaction with glucose levels, flexibility, and hypoglycemia. At visit 2, participants of the interventional group will additionally complete an adapted version of the Mobile Application Rating Scale (MARS) to assess the perceived quality of the intervention [39]. All primary and secondary outcomes are depicted in Fig. 2.

### Statistical analysis

All evaluations on positive care effects will base on intention-to-treat analyses. Missing values will be identified and replaced by using multiple imputation [40]. Additional sensitivity analysis will be performed with the per-protocol population. Descriptive data will be presented as absolute and relative frequencies (categorical, nominal data), median and interquartile range (75% percentile and 25% percentile; ordinal data), and mean ± SD (interval-scaled variables). The differences between the interventional and control groups will be assessed by the analysis of covariance with baseline data as a covariate, and follow-up data and group membership as dependent and independent variables, respectively. To control for multiplicity, analysis of primary and secondary endpoints will be carried out using a hierarchical testing approach with the following sequence: PAID, HbA1c, DSMQ, DES, percentage of hypoglycemic blood glucose values, number of blood glucose measurements per day, percentages of euglycemic and hyperglycemic values, average blood glucose levels, GSE, WHO-5, and DSat. For statistical analysis, SPSS 25 and Systat 12 will be used. All data will be treated confidentially and stored in pseudoanonymized form, anonymized when possible, and deleted after the end of the study. DE, NH, and BK will have access to the final trial dataset and will be responsible for analyses.

### Implementation and dissemination

When the efficacy of the digital health application can be demonstrated, permanent reimbursement within the German Digital Healthcare Act is pursued. With permanent reimbursement, widespread implementation of the application into clinical practice can be achieved. Furthermore, the results on the efficacy will be shared with health insurance companies as well as healthcare professionals to allow the widespread dissemination of results.

## Discussion

In this article, we present the study design of a multicenter, open-label, randomized, parallel-group, controlled trial to evaluate the health care effects and medical benefits of a smartphone-based diabetes self-management app. Outcome measures will comprise both direct medical benefits on blood glucose levels, HbA1c, and quality of life as well as patient-reported outcomes relevant for the health behavior of people with diabetes such as treatment adherence, patient autonomy, and coping with illness-related difficulties in everyday life.

We decided to initiate a prospective randomized, controlled study to potentially prove the causation of benefits and effects and to enhance evidence by randomization of patients and intention-to-treat analyses [41]. This study is intended to confirm the findings of a retrospective systematic analysis of 5920 mySugr PRO users (57.8% people with type 2 diabetes), as required by the Digital Healthcare Act. In this study, the largest group (*n* = 3836, initial eHbA1c < 7.5%) showed a stable eHbA1c of around 6.4% over 3 and 6 months. In the subgroup with initial eHbA1c > 9%, eHbA1c levels decreased by 1.5% and 1.7% after 3 and 6 months, respectively [27]. Since HbA1c analysis is generally accepted to provide evidence on the average blood glucose level during the previous 8 to 12 weeks [42], we concluded that the 12-week duration of the study period should be sufficient to demonstrate effectiveness. Furthermore, the criteria of the Digital Healthcare Act require the final study report to be completed within 12 months after the beginning of recruitment [26]. A longer follow-up period would therefore not be feasible. Likewise, the choice of the control group (treatment-as-usual) seems to be appropriate because no other digital application for diabetes self-management has been assessed in an approach meeting comparable scientific and methodological requirements so far.

As outcome measures, we will not only assess the direct medical effects on blood glucose and HbA1c levels because of their relationship with diabetic complications [43, 44] but also several PRO assessing psychological aspects of diabetes that have been shown to be important to people with diabetes [45, 46]. Preliminary evidence for the beneficial effects of mySugr on specific PRO was obtained in a retrospective systematic analysis, which was conducted during the application process for the Digital Healthcare Act. In this study, the comparison of 1099 study participants using the app with a historical cohort of 824 non-users revealed significantly decreased PAID-5 scores in app users with type 1 or type 2 diabetes (overall sample, 6.32 ± 4.36 vs. 5.07 ± 4.20, *t*(1921) = 6.36, *p* < 0.001; type 1 diabetes, 6.55 ± 4.30 vs. 5.12 ± 3.96, *t*(815) = 4.85, *p* < 0.001; type 2 diabetes, 6.01 ± 4.42 vs. 4.96 ± 4.23; *t*(1066) = 3.75, *p* < 0.001) [27]. Therefore, diabetes distress will be evaluated as the primary endpoint by the PAID questionnaire [33], a widely accepted scale to assess diabetes distress, since its results are associated with the functionality of coping styles, quality of life, and the occurrence of depressive symptoms [47]. Diabetes distress is common among people with diabetes, with a prevalence of approximately 20 to 40% [48, 49] and 36%, respectively [12]. Diabetes distress hinders the optimal management of diabetes and impacts on the emotional well-being, self-care, and quality of life of people with diabetes [13, 50] and, therefore, represents an important and valid outcome measure [51–53]. The broad spectrum of secondary endpoints then aims at reflecting the complexity of diabetes therapy and care that includes the self-management of dietary control, physical activity, and health care use, as well as diabetes-specific empowerment, self-efficacy, and satisfaction with current treatment. There is a great body of evidence that corresponding PRO are clinically relevant as they are associated with glycemic parameters [54], which is particularly true for PRO measures such as diabetes distress and self-management behaviors [13, 47, 50, 55].

We recognized the lack of blinding as a major limitation of our study design that might affect the generalizability of results. Obviously, blinding to reduce bias due to the knowledge of which intervention is being received is not possible in this setting. However, we believe that the impact of this limitation will be low for three reasons. First, the frequency of visits (beginning and end of study) prevents clinicians from reflecting their opinions on the allocation of participants and potential outcomes to people with diabetes. Second, the intervention will be used in the private environment of people with diabetes and independently from the health care provider. Third, outcomes will be correlated, for example, with the frequency of and the satisfaction with app usage, and the potential influence of these and other variables will be assessed by statistical mediation analysis.

In conclusion, this study aims to evaluate if diabetes distress can be reduced by the use of a smartphone-based diabetes self-management app (mySugr PRO) when compared to the control group. We will further assess if the use of the app supports the diabetes self-management of people with diabetes in several aspects relevant to medical and health care outcomes. In summary, this study will provide valuable insights into the efficacy of a digital health application in people with diabetes in a methodologically sound randomized controlled trial.

## Trial status

The protocol version number is 1.3, 21.12.2020. The protocol was amended to reflect the deletion of a questionnaire on physician satisfaction and inclusion of an updated version of the quality management system of the application. The recruitment began on February 11, 2021. Recruitment is expected to be completed on May 31, 2022, with the last-patient-last-visit at the end of August 2022.

## Data Availability

Not applicable; this is only a study protocol, and as such, no unpublished data are available.

## Abbreviations

BfArM: German Federal Institute for Drugs and Medical Devices (Bundesinstitut für Arzneimittel und Medizinprodukte)
CGM: Continuous glucose monitoring
DES: Diabetes Empowerment Scale
DiGA: Digital Health Application (digitale Gesundheitsanwendung)
DSat: Questionnaire for the assessment of diabetes-related problems and satisfaction with insulin treatment
DSMQ: Diabetes Self-Management Questionnaire
eHbA1c: Estimated HbA1c
GSE: General Self-Efficacy
MARS: Mobile Application Rating Scale
mEMA: Mobile ecological momentary assessment
PAID: Problem Areas In Diabetes
PRO: Patient-reported outcomes
